# Impact of Urethroplasty on Erectile Function: A Multicenter Analysis of the International Index of Erectile Function Score Changes Across Different Etiologies of Urethral Stricture

**DOI:** 10.3390/jcm14092936

**Published:** 2025-04-24

**Authors:** Mikołaj Frankiewicz, Łukasz Białek, Marta Rydzińska, Michał Skrzypczyk, Rafał Pęksa, Marcin Folwarski, Adam Kaftan, Jakub Krukowski, Adam Kałużny, Marcin Matuszewski, Trauma and Reconstructive Urology Working Party of the European Association of Urology Young Academic Urologists

**Affiliations:** 1 Department of Urology, Medical University of Gdańsk, 80-214 Gdańsk, Poland; adam.kaluzny@gmail.com (A.K.); matmar@gumed.edu.pl (M.M.); 2 Department of Urology, Centre for Postgraduate Medical Education, 01-004 Warsaw, Poland; lukaszbtm@gmail.com (Ł.B.); martaaga.drazkiewicz@gmail.com (M.R.); michalskrzypczyk@gmail.com (M.S.); 3 Department of Pathomorphology, Medical University of Gdansk, 80-214 Gdańsk, Poland; rafal.peksa@gumed.edu.pl; 4 Department of Clinical Nutrition and Dietetics, Medical University of Gdansk, 80-214 Gdańsk, Poland; 5 Home Enteral and Parenteral Nutrition Unit, Nicolaus Copernicus Hospital, 80-152 Gdańsk, Poland; 6 Faculty of Medicine, Medical University of Gdańsk, 80-214 Gdańsk, Poland; adamkaftan999@gmail.com; 7 LuxMed Medical Centre, 80-215 Gdańsk, Poland; krukowski.kuba@gmail.com

**Keywords:** urethral stricture, erectile dysfunction, urethroplasty, treatment outcome, international index of erectile function, postoperative period

## Abstract

**Background/Objectives**: Urethral stricture disease, characterized by narrowing of the urethra due to scar tissue, affects urinary and sexual health. While urethroplasty is the standard treatment, its impact on erectile function is less understood. This study examines changes in International Index of Erectile Function (IIEF) scores post-urethroplasty across various stricture etiologies, identifies predictors of erectile function outcomes, and explores recovery trajectories following surgery. **Methods**: This multicenter retrospective study included 103 patients who underwent urethroplasty between 2017 and 2023. Preoperative and postoperative IIEF scores at 3 and 6 or 12 months were analyzed. Stricture etiologies included pelvic fracture urethral injury, transurethral resection, catheterization, idiopathic, and hypospadias. The Wilcoxon signed-rank test and multivariate regression models were used to assess changes in IIEF scores and identify significant predictors. **Results**: Preoperative erectile function and patient age were significant predictors of postoperative outcomes. Younger patients and those with higher baseline IIEF scores experienced better erectile function post-surgery. Long-term outcomes (6 to 12 months) were significantly worse for strictures involving both penile and bulbar regions. Multivariate analysis showed higher pre-surgery IIEF scores and younger age were associated with better outcomes both short-term (R^2^ = 0.562) and long-term (R^2^ = 0.507). Diabetes was associated with worse erectile function outcomes at 3 months post-surgery. **Conclusions**: Younger patients and those with higher baseline IIEF scores have better erectile function outcomes following urethroplasty. Complex strictures involving both penile and bulbar regions adversely affect long-term outcomes. Additionally, the presence of diabetes is correlated with diminished erectile function in the short-term postoperative period.

## 1. Introduction

Urethral stricture disease, characterized by the narrowing of the urethra due to scar tissue formation, presents a significant clinical challenge with implications extending beyond urinary dysfunction to include sexual health. Urethroplasty provides the best long-term results and is still considered the gold standard treatment for this condition. Ongoing research investigates the impact of urethroplasty on sexual functions, including erectile, ejaculatory, and orgasmic dysfunctions, which are vital to patient quality of life. The International Index of Erectile Function (IIEF) score serves as a validated tool for the assessment of erectile function, providing a quantitative measure to evaluate changes post-urethroplasty. Studies have reported varying effects of urethroplasty on IIEF scores, with some patients experiencing a decline in erectile function postoperatively, while others demonstrate improvement or no significant change [1,2,3,4,5,6]. As the postoperative incidence of erectile dysfunction (ED) is likely influenced by multiple factors, there is a need to search for predictors of ED outcomes, including the role of patient-specific variables such as preoperative sexual function, psychological state, and precise surgical techniques used, to better tailor interventions and optimize patient outcomes. According to available research, 1% (0–38%) of patients experience sexual dysfunction following anterior urethroplasty, and 3% (0–34%) experience it following urethral damage repair associated with pelvic fractures [7,8,9].

Given the complexity of factors influencing sexual function after urethroplasty, this study aims to focus on ED and analyze the changes in IIEF scores at different time points before and after surgery across different etiologies of urethral stricture. By examining the relationship between the causes of stricture formation and ED, the study is designed to identify patterns that can guide patient counseling and surgical decisions for optimal urinary and sexual health outcomes. Moreover, the study evaluates the recovery trajectory of erectile function following surgical interventions in patients with varying severities of ED.

## 2. Materials and Methods

### 2.1. Study Design, Participants, and Data Collection

This multicenter retrospective study involved 103 patients undergoing urethroplasty between 2017–2023 in two reconstructive urology referral centers specialized in urethral surgery. The IIEF questionnaire uses rating scales ranging from 0 to 5 or 1 to 5 for its response options. The responses are summed to demonstrate a total IIEF score, which ranges from 1 to 25, with lower values indicating poorer sexual function. Erectile dysfunction is classified into five severity grades based on the IIEF score: absence of ED (22–25), mild (17–21), mild to moderate (12–16), moderate (8–11), and severe (1–7) [10,11].

Eligibility criteria for inclusion were adult male patients (aged ≥18 years) admitted for surgery due to urethral stricture, with available preoperative and postoperative IIEF scores at 3 months and either 6 or 12 months postoperatively. Patients with incomplete data or follow-up periods shorter than 6 months were excluded. Data on preoperative and postoperative IIEF scores were reported on printed questionnaires collected at four time points: preoperatively, at 3 and 6 to 12 months postoperatively. Additional data included patient age at surgery, comorbidities, etiology of the stricture, localization of the stricture, and the type of surgical procedure performed.

### 2.2. Ethics Statement

The study was conducted in accordance with the Declaration of Helsinki and approved by the institutional ethical committee (NKBBN/399/2017). Informed written consent was obtained from all participants, ensuring confidentiality and compliance with ethical standards.

### 2.3. Statistical Analysis

The dataset was processed using STATA statistical software (Version Stata/BE 18.0, StataCorp LLC, College Station, TX, USA). For each etiological subgroup, changes in IIEF scores were assessed using the Wilcoxon signed-rank test to compare preoperative scores with those obtained at subsequent follow-ups. This non-parametric test was chosen due to the non-normal distribution of score changes. Statistical significance was set at a *p*-value of less than 0.05.

## 3. Results

### 3.1. Study Group Characteristics

The mean age at the time of surgery was 48.0 years (range: 18–85 years). The etiologies of the strictures were diverse, with pelvic fracture urethral injury (PFUI) being the most common (40 patients, 38.8%), followed by catheterization (27 patients, 26.2%), transurethral resection of prostate or bladder tumor (TUR) (15 patients, 14.6%), idiopathic causes (11 patients, 10.7%), and hypospadias (10 patients, 9.7%). The strictures were predominantly located in the membranous urethra (40 patients, 38.8%), bulbar urethra (36 patients, 34.9%), with fewer cases in the penile (24 patients, 23.3%) and multi-site (penile and bulbar) urethra (3 patients, 2.9%). Various surgical techniques were employed, including bulbo-prostatic urethroplasty (BPA) in 40 patients (38.8%), end-to-end anastomosis in 33 patients (32.0%), buccal mucosa graft (BMG) in 15 patients (14.6%), and flap urethroplasty (preputial and skin flap combined) in 15 patients (14.6%). Combined study group characteristics are presented in Table 1. 

### 3.2. Analysis of Changes in IIEF Scores by Etiological Subgroup

The Wilcoxon signed-rank test was utilized to evaluate changes in IIEF scores preoperatively and at various follow-up periods across different etiological subgroups. The analysis revealed that for the Catheterization, Hypospadias, Idiopathic, and TUR subgroups, there were no statistically significant changes in IIEF scores between the preoperative and follow-up periods. In contrast, the Pelvic Fracture Urethral Injury (PFUI) subgroup exhibited a significant improvement in IIEF scores at 6–12 months. Notably, patients in this group typically experienced severe or moderate erectile dysfunction.

Statistical analysis was also conducted to determine if preoperative ED severity, measured by IIEF-5 scores, significantly differed among urethral stricture etiological subgroups. Although descriptive trends indicated that patients with PFUI tended to have lower median preoperative scores compared to other etiologies, the Kruskal–Wallis test revealed no statistically significant overall difference (*p* = 0.316). Subsequent pairwise comparisons using Mann–Whitney U tests with Bonferroni corrections similarly found no statistically significant differences between any two etiological groups. Thus, despite observed clinical variations, an association between preoperative erectile dysfunction severity and stricture etiology could not be statistically confirmed.

**Figure 1:**
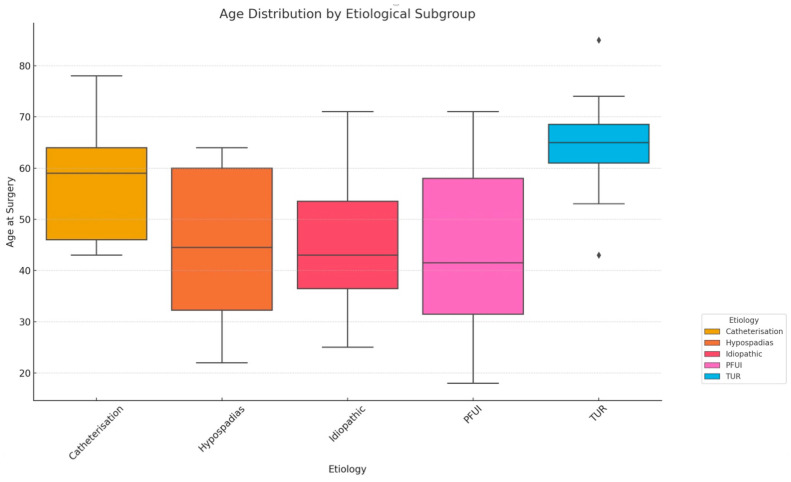
Age distribution in reference to etiology of the stricture. The plot depicts the age distribution of patients at the time of surgery across each etiological subgroup, highlighting the central tendency, variability, and outliers within each group.

Figure 1 presents a box-and-whisker plot illustrating the age distribution of patients undergoing urethroplasty, categorized by the etiological subgroup of their urethral strictures. Patients treated for strictures resulting from transurethral resection (TUR) procedures generally exhibit the highest median age, with a relatively narrow age distribution, indicating a consistently older patient population. Also, post-catheterization strictures were mostly presented by the older population. In contrast, patients with strictures due to PFUI have a notably wider age distribution, with a younger median age and more variability. The hypospadias and idiopathic subgroups show intermediate median ages.

**Figure 2:**
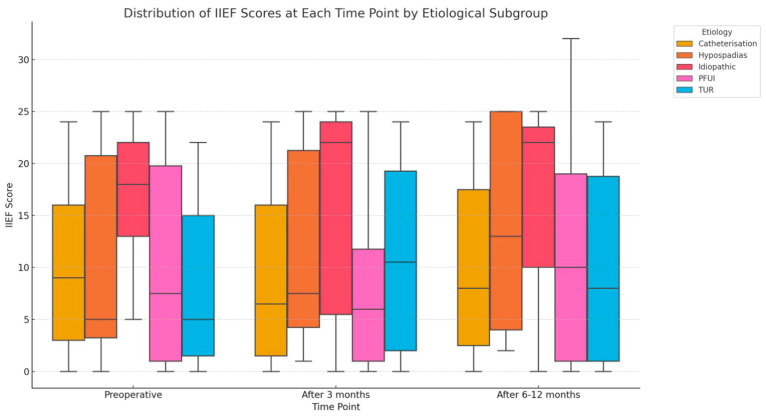
IIEF scores distribution in reference to etiology of the stricture. The plot illustrates the distribution of IIEF scores at various time points—preoperatively, at 3 months, and at 6–12 months post-surgery—across each etiological subgroup.

The graph in Figure 2 shows the distribution of International Index of Erectile Function (IIEF) scores across three different time points—preoperative, after 3 months, and after 6–12 months—categorized by the etiology of urethral strictures. Patients with pelvic fracture urethral injuries (PFUI) generally exhibit low median preoperative scores, suggesting severe erectile dysfunction initially. However, this subgroup notably demonstrates improvement at 6–12 months postoperatively. Patients with idiopathic strictures maintain relatively stable and higher median IIEF scores over the follow-up periods, indicating less severe erectile dysfunction preoperatively and consistent postoperative erectile function. The catheterization, hypospadias, and TUR groups display considerable variability, with minimal yet visible changes observed in median scores across time points. Overall, the graph highlights the variable impact of stricture etiology on erectile function recovery trajectories following urethroplasty.

### 3.3. IIEF Score Changes over Time by Erectile Dysfunction (ED) Level

**Figure 3:**
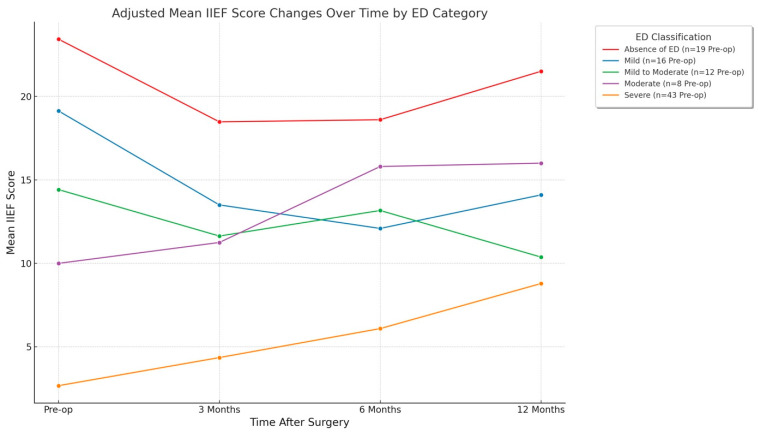
Adjusted mean IIEF score changes over time by ED level. This figure demonstrates the mean IIEF scores over time for patients stratified by preoperative ED severity, with the timeline encompassing four distinct points: preoperative, 3 months, 6 months, and 12 months post-surgery. Each line in the graph represents one ED level: absence of ED, (red line, n = 19), mild (blue line, n = 16), mild-to-moderate (green line, n = 12), moderate (violet line, n = 8), and severe (orange line, n = 43).

The adjusted mean IIEF score changes over time by ED level are depicted in Figure 3. This figure demonstrates the mean IIEF scores over time for patients stratified by preoperative ED severity, with the timeline encompassing four distinct points: preoperative, 3 months, 6 months, and 12 months post-surgery. Each line in the graph represents one ED level: absence of ED (n = 19), mild (n = 16), mild to moderate (n = 12), moderate (n = 8), and severe (n = 43). The results indicate that patients with severe ED exhibited improvement in mean IIEF scores, increasing from approximately 6 preoperatively to around 10 at 12 months post-surgery. Conversely, patients with no ED preoperatively had the highest mean scores (approximately 23), which slightly declined at 3 months, followed by stabilization and a slight increase by 12 months. Patients with mild and mild-to-moderate ED experienced a gradual decline in mean IIEF scores over time, whereas those with moderate ED showed some improvement, reflecting variability in surgical outcomes based on the initial severity of ED. This visualization highlights the differential impact of surgical interventions on erectile function across varying severities of preoperative ED.

### 3.4. Impact of Surgical Technique on Erectile Function Changes After Urethroplasty

The mean IIEF-5 scores were compared across different surgical methods at four time points (preoperative, 3, 6, and 12 months postoperatively). Buccal mucosa graft (BMG) urethroplasty showed stable mean scores with slight postoperative improvement, whereas bulbo-prostatic anastomosis (BPA) initially showed a decline at 3 months, followed by a moderate recovery by 6 months. End-to-end anastomosis and skin flap urethroplasty groups showed minimal fluctuations over time. However, Friedman’s tests indicated no statistically significant differences in IIEF-5 score changes within each surgical group over the postoperative periods (all *p*-values > 0.05), suggesting that the observed variations in erectile function across different urethroplasty techniques were not statistically significant.

### 3.5. Multivariate Model Analysis

The multivariate model analysis was conducted to further define the predictors of postoperative erectile function at both short-term (3 months) and long-term (6 or 12 months) intervals.

#### 3.5.1. Short-Term Model (3 Months Postoperatively)

The short-term model, which explained approximately 56.2% of the variance in IIEF scores (adjusted R-squared = 0.562), identified age at surgery and pre-surgery IIEF score as significant predictors of postoperative erectile function. Specifically, the model indicated that each additional year of age was associated with a 0.22-point decrease in the IIEF score (coefficient: −0.22, *p* < 0.001), suggesting that younger patients tend to have better erectile function outcomes shortly after surgery. Additionally, a higher baseline erectile function score was positively correlated with better short-term outcomes, with a coefficient of 0.57 (*p* < 0.001), indicating that each point increase in the baseline IIEF score corresponded to a 0.57-point increase in the short-term postoperative score. Notably, the type of surgery and the location of the stricture did not significantly impact short-term outcomes. The multivariate model analysis with the short-term data is presented in Table 2.

#### 3.5.2. Long-Term Model (6 or 12 Months Postoperatively)

The long-term model explained approximately 50.7% of the variance in IIEF scores (adjusted R-squared = 0.507). Similar to the short-term model, age at surgery and pre-surgery IIEF score remained significant predictors of long-term erectile function outcomes. The coefficient for age at surgery was −0.22 (*p* < 0.001), reinforcing the finding that younger patients achieve better long-term erectile function outcomes. The pre-surgery IIEF score had a coefficient of 0.50 (*p* < 0.001), signifying that higher baseline scores were associated with better long-term outcomes. Additionally, strictures located in both the penile and bulbar regions were found to have a significantly negative impact on long-term erectile function, with a coefficient of −15.88 (*p* = 0.017). This finding suggests that the anatomical complexity or extent of these strictures may adversely affect long-term recovery. The multivariate model analysis with the long-term data is presented in Table 3.

### 3.6. Impact of Comorbidities on Erectile Function Outcomes

To assess the influence of comorbidities on erectile function outcomes, multiple linear regression analysis was conducted, with the primary outcome being the IIEF score at 3 months and at 6 or 12 months postoperatively. The comorbidities included in the analysis were diabetes, hypertension, hyperlipidemia, coronary artery disease (CAD), chronic obstructive pulmonary disease (COPD), and smoking status. The regression models were adjusted for age and body mass index (BMI) and were evaluated for statistical significance using the F-statistic, with individual predictors assessed through *t*-tests. In the short-term model, the regression explained 11.1% of the variance in IIEF scores (R^2^ = 0.111, Adjusted R^2^ = 0.055), though the overall model did not reach statistical significance (F-statistic = 1.972, *p* = 0.0773). Notably, diabetes was identified as a significant predictor of poorer erectile function outcomes at 3 months post-surgery, with a coefficient of −7.47 (*p* = 0.031). Other comorbidities, including hypertension, hyperlipidemia, CAD, COPD, and smoking status, did not show statistically significant effects on erectile function at this time point.

In contrast, the long-term model, which explained 9.2% of the variance in IIEF scores (R^2^ = 0.092, Adjusted R^2^ = 0.036), did not identify any comorbidities as significant predictors of erectile function outcomes at 6- or 12-months post-surgery (F-statistic = 1.639, *p* = 0.144). These findings underscore the short-term negative impact of diabetes on erectile function recovery post-urethroplasty, while other comorbidities did not exhibit significant effects over the observed follow-up periods.

## 4. Discussion

The study’s findings indicate that postoperative erectile function following urethroplasty is influenced by several factors, including patient age, baseline erectile function, and the complexity of the urethral stricture. Younger patients and those with higher preoperative IIEF scores generally experience better outcomes at both 3 months and 6 or 12 months postoperatively. This aligns with previous research suggesting that age and baseline erectile function are critical determinants of recovery, as older patients often face age-related vascular and neurological challenges that can impede recovery [1]. 

The study also highlights the significant impact of diabetes on short-term erectile function, underscoring the importance of managing this condition preoperatively to optimize outcomes. In our study, diabetes was identified as a significant predictor of poorer erectile function outcomes at 3 months post-surgery. This finding is consistent with existing literature that emphasizes the essential role of diabetes in vascular health and erectile function [12,13,14]. 

A study by Kouidrat et al. highlights that ED is a common complication of diabetes, affecting more than half of men with the condition. The prevalence of ED in diabetic men is approximately three times higher than in non-diabetic men, and it increases with the duration and poor control of diabetes. This is due to the multifactorial nature of diabetic complications, including macroangiopathy, microangiopathy, and neuropathy, all of which contribute to ED. Furthermore, diabetes-related vascular damage can impair vasodilation and lead to conditions such as atherosclerosis, which can further impair erectile difficulties [15,16]. Given these factors, managing diabetes preoperatively is crucial not only to improve patients’ recovery and ensure optimal conditions for healing of the urethra but also for optimizing erectile function outcomes post-urethroplasty. Addressing diabetes can help mitigate its negative impact on vascular health, potentially improving the recovery trajectory of erectile function following surgical interventions. 

Interestingly, a study by Mondal et al. found no significant change in vascular parameters like peak systolic velocity and resistive index of the cavernosal artery after urethroplasty, suggesting that the surgical procedure per se may not result in ED. The authors concluded that, while there is a significant prevalence of ED in patients with urethral strictures, the postoperative incidence of ED after urethroplasty is likely multifactorial and not solely attributable to the surgery itself [3]. This theory may be supported by the results of Xie and colleagues, who evaluated ED following graft urethroplasty for complex urethral strictures (>10 cm long, at one or more sites). Of 65 eligible patients, 46.15% (30 patients) had normal erectile function before surgery, with 36.92% (24 patients) maintaining it three months post-surgery. Of the 20 patients with erectile dysfunction due to pelvic fracture or iatrogenic trauma, 45% (9 patients) regained potency three months post-surgery, with no changes at six months. No significant differences in ED were observed between patients with anterior and multi-site strictures before surgery, but significant differences were noted at 3-, 6-, and 12-months post-surgery (*p* < 0.05) [17]. 

Zhao et al. demonstrated that non-transecting bulbar urethroplasty is associated with a significantly lower risk of erectile dysfunction compared to transecting approaches, without compromising stricture resolution rates [18]. Moreover, Shalkamy et al. reported that permanent de novo erectile dysfunction is uncommon and occurs at comparable rates between transecting and non-transecting techniques [19]. 

Interestingly, in a recent study, Mousa et al. found superior erectile outcomes with ventral versus dorsal buccal mucosal graft onlay in proximal bulbar urethroplasty, likely due to reduced disruption of perineal structure [20]. These findings support a growing consensus that technical modifications during urethroplasty may mitigate adverse sexual outcomes. 

Dogra et al. described in their literature review that anterior urethroplasty, specifically transecting bulbar urethroplasty, carries a higher risk of sexual dysfunction compared to non-transecting or penile urethroplasty [21]. This suggests that the surgical technique and the specific location of the urethral stricture can significantly influence postoperative sexual outcomes. On the other hand, some studies suggest that urethroplasty itself does not significantly affect erectile function. Erickson et al. found that urethral reconstructive surgery positively influenced ejaculatory functions without significantly affecting erectile function or causing sexual dysfunction. However, older men reported experiencing more ED after the surgery compared to younger men, although their erectile function likely improved over time [22]. 

Eltaher et al. add to this by offering a focused analysis of the transient nature of ED post-urethroplasty and its variation with different surgical techniques [23]. The observations regarding erectile function post-bulbo-prostatic anastomosis (BPA) suggest that improvements in erectile quality may not be directly attributable to the surgical intervention itself. Instead, the delayed enhancement in erectile function observed beyond six months post-surgery could be linked to the natural neurovascular regeneration processes rather than the reconstructive surgery, which should not inherently affect structures responsible for erections. The observed improvements in our PFUI subgroup underscore the potential for recovery in this demographic, emphasizing the importance of patient age as a factor that influences the chance for tissue regeneration. Although, it should be emphasized that patients in this group should always be counseled on the risk for persistent erectile dysfunction if present preoperatively. This information is crucial for managing postoperative expectations. Additionally, Johnson and Latini’s study contributes to understanding the minimal impact of urethroplasty on erectile function, as measured by validated questionnaires [24]. 

While in our study, the type of surgical technique and the specific location of the stricture did not significantly affect short-term outcomes, a negative association was observed between complex (penile + bulbar) strictures and long-term erectile function outcomes. However, given that only three patients (3%) in the cohort presented with this stricture configuration, this finding should be interpreted with caution and considered as a potential correlation rather than a definitive causal relationship. Nonetheless, the anatomical complexity and surgical trauma in these cases likely contribute to disruptions in erectile function. This observation is supported by other studies that have noted similar challenges in managing complex strictures [2,3]. 

These findings have important implications for clinical practice. They emphasize the need for personalized treatment strategies that consider individual patient characteristics, such as age, baseline erectile function, and comorbidities, with special emphasis on diabetes to optimize postoperative outcomes. Preoperative counseling should address these factors to help patients set realistic recovery expectations. Additionally, recognizing stricture location as a determinant of long-term outcomes underscores the necessity for meticulous surgical planning, particularly in complex cases involving multiple urethral regions. The anatomical complexity and extent of urethral strictures likely contribute to more significant disruptions in erectile function, possibly due to greater surgical manipulation and trauma to the surrounding tissues. 

A key unresolved question is whether postoperative erectile dysfunction arises from the urethral stricture itself or the surgical repair. While urethroplasty may contribute to neurovascular disruption, many patients present with preexisting dysfunction related to the stricture etiology. This distinction remains critical yet difficult to delineate, underscoring the need for further research. Further studies in this field would also be valuable to determine if a step-by-step approach, involving staged surgical interventions, could improve sexual outcomes by minimizing trauma and allowing for better tissue healing.

This study has several limitations that should be considered when interpreting the results. First, the sample size for certain subgroups, particularly those classified by specific stricture locations and surgical methods, was relatively small, limiting the generalizability and statistical power to detect differences between these subgroups. Second, the follow-up period varied among patients, with data points collected at 6 and 12 months postoperatively, potentially introducing bias as recovery timelines may differ. A standardized follow-up period could provide more consistent and comparable results. Moreover, reliance on the IIEF score as the sole measure of erectile function might not capture the full scope of a patient’s sexual health and quality of life. The IIEF, while widely used and validated, does not encompass all aspects of sexual function, such as partner satisfaction and psychological impacts. Finally, the retrospective nature of the study could introduce selection bias and limit the ability to establish causal relationships. Prospective studies with randomized controlled designs are needed to confirm these findings and better understand the causal mechanisms.

## 5. Conclusions

Preoperative erectile function and patient age are significant predictors of postoperative outcomes. Younger patients and those with higher baseline IIEF scores tend to experience better short-term and long-term erectile function post-surgery. Additionally, diabetes is a significant predictor of poorer short-term erectile function outcomes, emphasizing the importance of managing this condition preoperatively to optimize recovery. The type of surgical method and the location of the stricture are not significant predictors of short-term outcomes. However, in the long term, complex strictures involving both penile and bulbar regions were associated with significantly worse erectile function outcomes. Future research should aim to validate these findings through larger, prospective studies and explore additional factors that may influence long-term sexual health and quality of life post-surgery.

## Figures and Tables

**Table 1:** Combined patient demographics and stricture characteristics.

Patient Variables	Total (%)
Age (years), median (min–max)	48 (18–85)
BMI, median (min–max)	27.6 (18–37)
Stricture length (cm), median (min–max)	2 (1–3)
Medical co-morbidities	
Current or former smokers	28 (26.7%)
Diabetic	12 (11.4%)
Hypertension	29 (27.6%)
Hyperlipidemia	16 (15.2%)
COPD or CAD	11 (10.5%)
Stricture Location	
Bulbar	36 (34.9%)
Penile	24 (23.3%)
Membranous	40 (38.8%)
Penile + Bulbar	3 (2.9%)
Etiology	
PFUI	40 (38.8%)
TUR	15 (14.6%)
Catheterisation	27 (26.2%)
Idiopathic	11 (10.7%)
Hypospadias	10 (9.7%)
Surgical method	
BPA	40 (38.8%)
End-to-end anastomotic urethroplasty	33 (32.0%)
BMG	15 (14.6%)
Skin flap urethroplasty	15 (14.6%)

**Table 2:** Multivariate model analysis: short-term model (3 months postoperatively).

Predictor	Coefficient	*p*-Value	95% Confidence Interval
Intercept	15.85	<0.001	[10.02, 21.69]
Localization (membranous)	−2.33	0.699	[−14.24, 9.58]
Localization (penile)	4.13	0.206	[−2.31, 10.57]
Localization (penile + bulbar)	6.03	0.326	[−6.11, 18.16]
Surgical method (BPA)	−4.43	0.484	[−16.94, 8.09]
Surgical method (end-to-end)	−2.35	0.222	[−6.16, 1.45]
Surgical method (flap)	−7.75	0.079	[−16.49, 1.0]
Age at surgery	−0.22	<0.001	[−0.3, −0.13]
IIEF (pre-surgery score)	0.57	<0.001	[0.41, 0.73]

**Table 3:** Multivariate model analysis: long-term model (6 or 12 months postoperatively).

Predictor	Coefficient	*p*-Value	95% Confidence Interval
Intercept	20.31	<0.001	[14.11, 26.51]
Localization (membranous)	−4.43	0.494	[−17.25, 8.39]
Localization (penile)	2.06	0.554	[−4.84, 8.97]
Localization (penile + bulbar)	−15.88	0.017	[−28.91, −2.85]
Surgical method (BPA)	−2.76	0.684	[−16.2, 10.68]
Surgical method (end-to-end)	−2.74	0.173	[−6.7, 1.22]
Surgical method (flap)	−8.32	0.055	[−16.87, 0.24]
Age at surgery	−0.22	<0.001	[−0.3, −0.13]
IIEF (pre-surgery score)	0.50	<0.001	[0.35, 0.65]

## Data Availability

The raw data supporting the conclusions of this article will be made available by the authors on request.

## Abbreviations

The following abbreviations are used in this manuscript:

BMG	Buccal mucosa graft
BMI	Body mass index
BPA	Bulbo-prostatic anastomosis
CAD	Coronary artery disease
CI	Confidence interval
COPD	Chronic obstructive pulmonary disease
ED	Erectile dysfunction
IIEF	International Index of Erectile Function
PFUI	Pelvic fracture urethral injury
TUR	Transurethral resection
